# Abnormal Enhancement of Protein Disulfide Isomerase-like Activity of a Cyclic Diselenide Conjugated with a Basic Amino Acid by Inserting a Glycine Spacer

**DOI:** 10.3390/biology10111090

**Published:** 2021-10-24

**Authors:** Rumi Mikami, Shunsuke Tsukagoshi, Kenta Arai

**Affiliations:** Department of Chemistry, School of Science, Tokai University, Kitakaname, Hiratsuka-shi 259-1292, Japan; 1cskm008@mail.u-tokai.ac.jp (R.M.); s-tsukagoshi@fuji.tokai-u.jp (S.T.)

**Keywords:** oxidative folding, enzyme model, selenium, aggregation, chaperone, catalyst

## Abstract

**Simple Summary:**

A polypeptide chain, which is a biological polymer composed of amino acids, can have a physiological function as a “protein” by forming a unique three-dimensional structure through protein folding. Structural failure of proteins in cells frequently induces various diseases, such as neurodegenerative diseases and diabetes. Therefore, cells are equipped with various enzymes, such as protein disulfide isomerase (PDI), to promote correct folding and avoid misfolding. In both cells and test tubes, protein folding is a key process in determining the overall efficiency of protein synthesis. Therefore, it is crucial to develop an artificial folding catalyst, which behaves like PDI, for both drug discovery and protein engineering. Herein, we report the structural optimization of a cyclic diselenide molecule to improve its intrinsic PDI-like catalytic activity.

**Abstract:**

In a previous study, we reported that (*S*)-1,2-diselenane-4-amine (**1**) catalyzes oxidative protein folding through protein disulfide isomerase (PDI)-like catalytic mechanisms and that the direct conjugation of a basic amino acid (Xaa: His, Lys, or Arg) via an amide bond improves the catalytic activity of **1** by increasing its diselenide (Se–Se) reduction potential (*E*′°). In this study, to modulate the Se–Se redox properties and the association of the compounds with a protein substrate, new catalysts, in which a Gly spacer was inserted between **1** and Xaa, were synthesized. Exhaustive comparison of the PDI-like catalytic activities and *E*′° values among **1**, **1**-Xaa, and **1**-Gly-Xaa showed that the insertion of a Gly spacer into **1**-Xaa either did not change or slightly reduced the PDI-like activity and the *E*′° values. Importantly, however, only **1**-Gly-Arg deviated from this generality and showed obviously increased *E*°′ value and PDI-like activity compared to the corresponding compound with no Gly spacer (**1**-Arg); on the contrary, its catalytic activity was the highest among the diselenide compounds employed in this study, while this abnormal enhancement of the catalytic activity of **1**-Gly-Arg could not be fully explained by the thermodynamics of the Se–Se bond and its association ability with protein substrates.

## 1. Introduction

**Figure 1:**
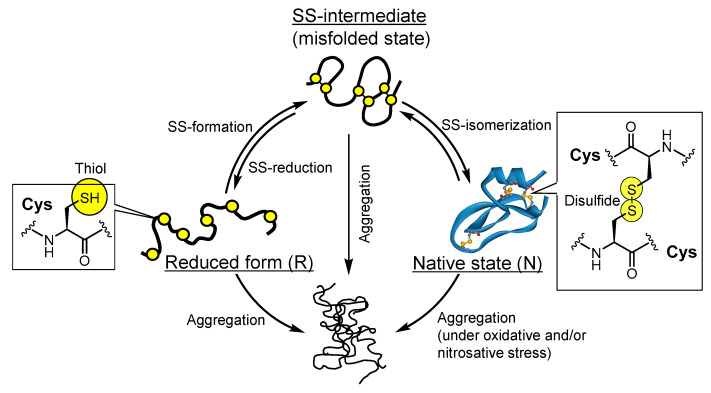
Disulfide-related reactions (SS-formation, SS-isomerization, and SS-reduction) and protein aggregation during oxidative folding.

Cross-linking of disulfide (SS) linkage(s) between cysteine (Cys) residues is a representative post-translational modification of nascent polypeptide chains to form a unique three-dimensional structure in cells [1,2]. Similarly, polypeptides artificially prepared by recombinant technology and chemical synthesis require structural maturation processes coupled with the formation of SS bonds, so-called oxidative folding, in a test tube to exert their biological functions. Typically during oxidative folding, reduced protein (R) with no SS bond gains non-native (mispaired) SS bonds via SS-formation, and the produced SS-intermediates subsequently rearrange the incorrect SS pairing into the correct pattern that is found in the native state (N) via SS-isomerization (Figure 1) [2]. Cleavage of SS bonds (SS-reduction) in proteins promotes the refolding reaction of the misfolded state and denaturation and degradation of overexpressed proteins and thus is an essential chemical reaction in intracellular protein quality control. However, the structurally immature proteins readily contact and combine with each other, resulting in the formation of insoluble oligomers and aggregates, which prohibit correct protein folding in test tubes as well as cells. Furthermore, many groups have reported that the formation of aggregates causes undesired cell death and various human diseases such as neurodegenerative disorders [3,4,5,6] and that the N form can also be converted into pathogenic oligomers and aggregates under oxidative and nitrosative stress [7,8,9,10]. Thus, from the viewpoints of both disease treatment (or prevention) and improvement in efficiency of chemical synthesis of proteins, it is important to develop chemical reagents and catalysts that can suppress aggregate formation while efficiently promoting SS-related reactions (Figure 1) both in cells and in test tubes. Therefore, many research groups, including ours, have addressed the development of artificial compounds that can promote correct oxidative folding [11,12,13,14,15,16,17,18,19].

**Figure 2:**
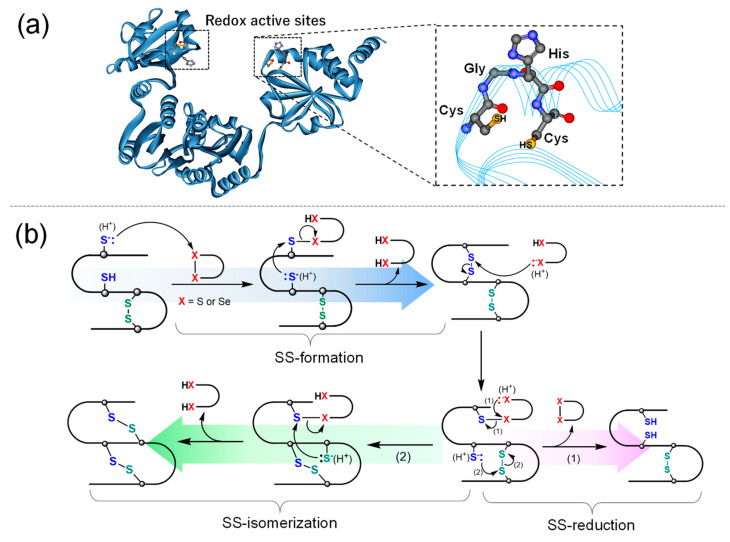
Protein disulfide isomerase (PDI). Overall three-dimensional structure and the active center of PDI (PDB code: 2b5e) (**a**) and its catalytic mechanisms for the SS-related reactions in substrate proteins (**b**).

**Scheme 1:**
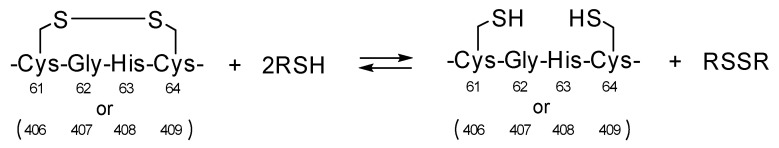
Reversible SS-SH redox reaction of Cys-Gly-His-Cys redox active sites in PDI.

In the biosynthesis of proteins, oxidative folding proceeds in the endoplasmic reticulum (ER), and protein disulfide isomerase (PDI), which is the most representative ER-resident oxidoreductase, catalyzes SS-related reactions, consequently promoting structural maturation of nascent polypeptide chains [20,21,22,23,24]. In addition, PDI possesses a chaperone-like activity, by which it suppresses protein aggregation. PDI consists of four tandem thioredoxin-like domains (i.e., a-b-b′-a′), and the a and a′ domains include the quartet sequence, Cys-Gly-His-Cys (CGHC), as a redox active center (Figure 2a). CGHC exists in two states, disulfide ([S–S]) and dithiol ([SH,SH]) forms, owing to the redox equilibrium between cysteinyl thiols and disulfides in the presence of substrate thiols and disulfides (RSH/RSSR), such as proteins and glutathione, as shown in Scheme 1. Consequently, [S–S] promotes SS formation via an intermolecular bond exchange reaction, whereas [SH,SH] catalyzes SS-isomerization and SS-reduction (Figure 2b).

The SH groups in the active sites of PDI have lower p*K*_a_ values (ca. 6.7) than the general cysteinyl SH groups (ca. 8.3) in proteins because of the effect of neighboring His63 (or His408) as a general base catalyst and therefore exhibit high nucleophilicity under biological pH conditions. Furthermore, the active site possesses a higher disulfide reduction potential (ca. −180 mV) [25] compared to the general cysteinyl disulfide in proteins, meaning that the [S−S] form functions as a strong SS-forming reagent for the substrate proteins, and the active site can dominantly exist in the [SH,SH] form under ER conditions. This further indicates that the [SH,SH] form can favorably promote SS-isomerization (path (2) in Figure 2b) rather than SS-reduction (path (1) in Figure 2b) due to the thermodynamic property of the [SH,SH] form, which is less likely to cyclize to the [S–S] form.

**Figure 3:**
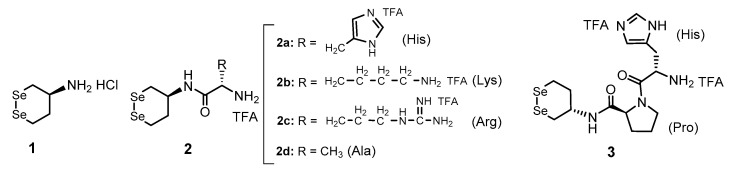
Diselenide compounds reported previously.

In recent years, we have reported that water-soluble cyclic diselenides such as (*S*)-1,2-diselenane-4-amine (**1**) [26] and its derivatives have intrinsic capabilities to mimic the functions of various oxidoreductases, including PDI [16,18,27,28]. Diselenide (Se–Se) is a covalent bond in which sulfurs in an SS bond are replaced with selenium, which is homologous to sulfur but a higher-period element and shows a redox behavior similar to that of SS bond, as shown in Scheme 1. Namely, the cyclic diselenide [Se–Se] can reversibly be converted into the corresponding diselenol [SeH,SeH] form by coexisting thiols (RSH), thus promoting SS-related reactions during oxidative folding via similar molecular mechanisms of PDI, as shown in Figure 2b. Since the selenol (SeH) group has a significantly lower p*K*_a_ value than the SH group, [SeH,SeH] exists almost as nucleophilic selenolate anions (Se^−^) in a physiological environment, and the coexistence of a catalytic amount of the active [SeH,SeH] in the oxidative folding can sufficiently promote SS-reduction and SS-isomerization [16,18]. However, the reductive activation of the Se–Se bond using a thiol (i.e., [Se-Se] + 2RSH → [SeH,SeH] + RSSR) is thermodynamically unfavorable because the reduction potentials of S–S compounds are generally much higher than those of Se–Se compounds. We previously suggested that by conjugating a basic amino acid with diselenide **1** as a parent compound, [SeH,SeH] produced by the reduction of [Se–Se] in situ is thermodynamically stabilized via the NH···Se hydrogen bond formed between the selenium atom and the basic amino acid side chain, meaning that the Se–Se reduction potential (*E*°′_SeSe_) of compounds **2a**–**d** (Figure 3) was significantly increased compared to that of compound **1** (−368 mV) (Table 1) [18]. In particular, conjugation of His increased the *E*°′_SeSe_ value of **1**, causing the greatest enhancement in the oxidative folding velocity. Furthermore, it was found that **2a** (**1**-His) remarkably suppressed protein aggregation even at a low concentration (≈ 0.3 mM), implying that the conjugation of a basic amino acid may also create a chaperone-like activity in the cyclic diselenide. Furthermore, by inserting a proline (Pro) spacer, a turn-promoting imino acid, between compound **1** and His, a rigid γ-turn structure was formed in the [SeH,SeH] state derived from compound **3**, resulting in the imidazole ring of His residue topologically approaching the selenium atom to possibly form an NH···Se hydrogen bond and further increasing the *E*°′_SeSe_ value (−289 mV) of **3** compared to **2a** with no Pro spacer [28]. Unexpectedly, however, the catalytic ability of **3** for oxidative folding was comparable to that of **2a**, despite the superiority of **3** over **2a** in *E*°′_SeSe_ value. This implies that steric hindrance due to the formation of a rigid foldamer may prohibit the contact of the reactive SeH groups with S–S linkages in proteins.

Thus, by creating appropriate flexibility in dipeptide-conjugated cyclic diselenide compounds while maintaining a high *E*°′_SeSe_ value, the ability of the compound as an oxidative folding catalyst may be further enhanced owing to the improvement in the contact frequency of the compound with substrate proteins. Therefore, in this study, we designed and synthesized compounds (**4**) with a glycine (Gly) spacer, which has the smallest side chain and provides flexibility to the peptide backbone as opposed to Pro, between the cyclic diselenide and a basic amino acid as a terminus (Table 1). To quantitatively investigate the utility of the compounds in oxidative folding, *E*°′_SeSe_ values, catalytic capabilities for the SS-related reactions (Figure 1), and suppressive abilities against protein aggregation were evaluated. Herein, we demonstrate an optimized structure of a cyclic diselenide-based compound as a PDI-like redox catalyst.

## 2. Materials and Methods

### 2.1. General

Melting points were measured with a Yanaco MP-S3 micro melting point system and were uncorrected. ^1^H (500 MHz), ^13^C (125.8 MHz), and ^77^Se (95.4 MHz) NMR spectra were recorded using a Bruker AV-500 spectrometer at 298 K, and the coupling constants (*J*) are reported in Hz. High-resolution mass spectra (HRMS) were recorded using a JEOL JMS-T100LP mass spectrometer under electrospray ionization (ESI+) conditions. Gel permeation chromatography (GPC) was performed using a JAI LC-918 high-performance liquid chromatograph (HPLC) system with CHCl_3_ as the eluent. Compounds **1** [16], **2a**–**d** [18], **3** [28], **4a**,**d** [28], and **1**-Gly [28] were synthesized according to the literature. Hen egg white lysozyme (HEL), GSH, GSSG, and DL-dithiothreitol (DTT^red^) were purchased at biological grade from FUJIFILM Wako Pure Chemical Corporation. The reduced form of HEL (R^HEL^) was prepared as previously described [29]. Briefly, native HEL (12 mg) was incubated at pH 8.0 and 40 °C for 2 h in 100 mM Tris–HCl/1 mM EDTA buffer solution containing 8 M urea and excess amount of DTT^red^ (12 mg) as a reductant. The resulting solution was desalted by passing through the column packed with Sephadex G25 resin, and the fraction containing a protein was collected and lyophilized to obtain a white powder of R^HEL^. The buffer solutions used in the folding experiments were fully purged with argon gas by bubbling for 30 min. All other chemicals and enzymes were used as purchased without further purification.

### 2.2. General Procedure for Synthesis of ***4b*** and ***4c***

**Scheme 2:**
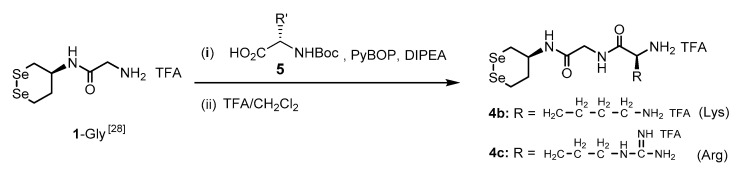
Synthesis of compounds **4b** and **4c** from **1**-Gly.

The target compounds **4b** and **4c** were prepared as shown in Scheme 2. Boc-L-amino acid (**5**, 2 eq) was added to a solution of **1**-Gly [28] dissolved in anhydrous DMF (1 mL). (Benzotriazol-1-yloxy)tripyrrolidinophosphonium hexafluorophosphate (PyBOP) (2 eq) and *N*,*N*-diisopropylethylamine (DIPEA) (4 eq) were added to the solution. The mixture was stirred for 14 h at 25 °C under an argon atmosphere. To remove impurities and DMF solvent, the mixture was diluted with 1 mL of EtOAc/*n*-hexane (5:1 [*v*:*v*]), the resulting solution was directly purified by silica gel column chromatography using EtOAc/*n*-hexane (5:1 [*v*:*v*]) as the eluent, and the fractions containing a Boc-protected dipeptide conjugate were collected, combined, and evaporated. The obtained yellow oil was further purified by GPC, and the collected fraction containing the Boc-protected compound was evaporated. The obtained residual material was dissolved in CH_2_Cl_2_, and TFA (0.5 mL) was added to this solution at 0 °C. After stirring at 0 °C for 30 min, the reaction temperature was increased to 25 °C, and the mixture was further stirred for 3.5 h while maintaining the temperature. The resulting solution was concentrated to remove the solvent and TFA under vacuum, and the dipeptide conjugate (**4b** and **4c**) was finally collected as a yellow solid.

### 2.3. (. S)-N-(2-(((S)-1,2-Diselenan-4-Yl)Amino)-2-Oxoethyl)-2,6-Diaminohexanamide di-TFA Salt (***4b***)

Using **1**-Gly (19.2 mg, 48.0 μmol) and Boc-L-lysine (i.e., R′ = -CH_2_-CH_2_-CH_2_-CH_2_-NHBoc in **5**) (33.9 mg, 96.0 μmol) and purifying the crude product of the Boc-protected compound using column chromatography, **4b** was finally obtained as a yellow solid. Yield: 25.2 mg (48%); m.p. 100.3–102.7 °C; ^1^H NMR (500 MHz, D_2_O): δ, 3.95–3.90 (m, 2H), 3.87–3.79 (m, 2H), 3.23–3.11 (m, 2H), 2.98–2.85 (m, 4H), 2.18–2.13 (m, 1H), 1.86–1.76 (m, 3H), 1.62–1.56 (m, 2H), 1.41–1.31 (m, 2H) ppm; ^13^C NMR (125.8 MHz, D_2_O): δ 170.2, 169.1, 52.9, 48.7, 42.3, 38.9, 34.3, 30.2, 26.3, 26.2, 23.9, 21.1 ppm; ^77^Se NMR (95.4 MHz, D_2_O): δ 264.1 ppm; HRMS (ESI-TOF) *m*/*z*: [M+H–2TFA]^+^ calculated for C_12_H_25_N_4_O_2_Se_2_^+^, 417.0302; found, 417.0306.

### 2.4. (. S)-N-(2-(((S)-1,2-Diselenan-4-Yl)Amino)-2-Oxoethyl)-2-Amino-5-Guanidinopentanamide di-TFA Salt (***4c***)

Using **1**-Gly (21.6 mg, 53.9 μmol) and Boc-L-arginine (i.e., R′ = -CH_2_-CH_2_-CH_2_-NH-C(=NHBoc)-NHBoc in **5**) (52.8 mg, 108 μmol) and purifying the crude product of a Boc-protected compound using column chromatography, **4c** was finally collected as a yellow solid. Yield: 23.2 mg (63%); m.p. 102.2–104.3 °C; ^1^H NMR (500 MHz, D_2_O): δ 3.94–3.87 (m, 2H), 3.81 (q, *J* = 12.0 Hz, 2H), 3.20–3.11 (m, 2H), 3.09 (t, *J* = 6.9 Hz, 2H), 2.95–2.84 (m, 2H), 2.15–2.10 (m, 1H), 1.86–1.74 (m, 3H), 1.61–1.50 (m, 2H) ppm; ^13^C NMR (125.8 MHz, D_2_O): δ 170.0, 169.1, 156.7, 52.8, 48.7, 42.3, 40.3, 34.3, 27.8, 26.2, 23.9, 23.4 ppm; ^77^Se NMR (95.4 MHz, D_2_O): δ 264.6 ppm; HRMS (ESI-TOF) *m*/*z*: [M+H–2TFA]^+^ calculated for C_12_H_25_N_6_O_2_Se_2_^+^, 445.0364; found, 445.0358.

### 2.5. Heat-Denaturation-Induced Aggregation of Hen Egg-White Lysozyme

Heat denaturation of HEL was carried out according to our previous protocol, with slight modifications [18]. Briefly, 800 μL of 50 mM Tris-HCl buffer solution containing 100 μM native HEL, 0 or 0.5 mM diselenide compound, 0.5 mM EDTA, and 0.3 M NaCl at pH 7.5 was heated in a dry thermo bath. The temperature was increased in a stepwise manner from 30 to 50 °C, 50 to 70 °C, 70 to 80 °C, and 80 to 90 °C, and the sample solutions were incubated for 5 min at each temperature. After cooling the sample solution to 23 °C, 200 μL of aliquot was transferred to a 96-well plate, and light scattering at 620 nm was measured using a microplate reader (Multiskan FC; Thermo Fisher Scientific K.K., Minato, Japan).

## 3. Results and Discussion

### 3.1. Synthesis of Gly-Xaa Dipeptide-Conjugated ***1*** (***1***-Gly-Xaa)

While Gly residue generally imparts flexibility to the peptide backbone, it is also frequently found as a component amino acid in turn structures of proteins. Thus, the Gly spacer may also assist in the topological approach of a terminal basic amino acid to the selenium atom to stabilize the [SeH,SeH] form via an NH···Se hydrogen bond, while preserving the appropriate flexibility of the peptide backbone. Therefore, compounds with a Gly spacer, **4a** (**1**-Gly-His) [28], **4b** (**1**-Gly-Lys), and **4c** (**1**-Gly-Arg), were prepared as analogs of the basic amino acid conjugates, **2a** (**1**-His), **2b** (**1**-Lys), and **2c** (**1**-Arg), respectively (see Section 2 for experimental details). As a result, the target compounds were successfully obtained in reasonable yields from **1**-Gly [28] as a precursor.

### 3.2. Diselenide Reduction Potential of Compounds

**Figure 4:**
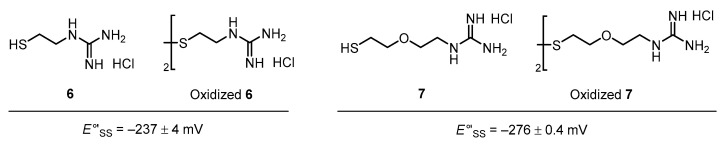
Monothiol compounds **6** and **7** and their oxidized forms with a guanidyl unit.

First, the compounds’ Se–Se reduction potential (*E*°′_SeSe_), which is one of the most important determinants of the capability of a compound as an oxidative folding catalyst, was measured by following our previous protocol (Table 1) [16]. Both the compounds with and without Gly spacer, i.e., series **2** and **4**, respectively, were found to possess higher *E*°′_SeSe_ values than that of the parent compound (**1**), suggesting that the open-chain diselenols were thermodynamically stabilized in each case, possibly by a weak interaction such as an intramolecular NH···Se hydrogen bond between the side chain of the basic amino acid and the selenium atom. The results further showed a general trend that the insertion of a Gly spacer between **1** and the basic amino acids decreased or did not change the *E*°′_SeSe_ value. Muraoka et al. previously reported that thiol compound **6** and its disulfide form (oxidized **6**), which are covalently conjugated with a guanidyl group, have a higher *E*′°_SS_ value (*E*′°_SS_ = −237 mV) than glutathione (GSH; *E*′°_SS_ = −256 mV [30]), which is used as a common oxidative folding reagent; hence, by adding **6** and oxidized **6** rather than adding GSH and its oxidized form (GSSG), respectively, the oxidative folding efficiency is improved in terms of both the rate and yield [17] (Figure 4). Recently, their group reported that insertion of a diethylene glycol moiety as a spacer motif between the thiol and guanidyl unit decreases the *E*′°_SS_ value, reducing its activity as an oxidative folding promoter [19]. This is likely because of the increased distance between the sulfur atom and charged guanidyl unit, resulting in a weaker thiol-stabilizing interaction, such as an electrostatic interaction (NH^+^···S^−^) and/or NH···S hydrogen bonds [31]. Thus, our observations in the reduction potential measurements are generally consistent with these previous results.

**Figure 5:**
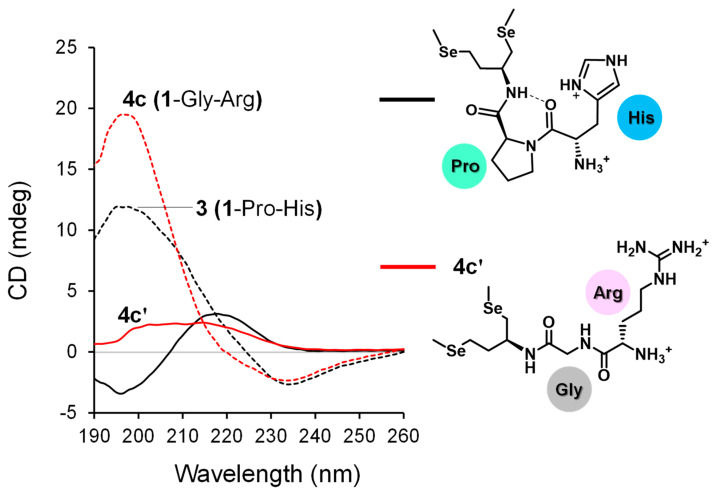
CD spectra of compounds (200 μM) in 10 mM Tris-HCl buffer solution containing 1 mM EDTA at pH 7.5 and 25 °C. The spectra were determined using a quartz cell (path length = 1 mm). Data of **3** (black dashed line) and its open-chain form (black solid line) were reported previously [28].

Interestingly, however, insertion of the Gly spacer abnormally increased the *E*°′_SeSe_ value of the compound only for Arg-conjugated compounds (**2c** [**1**-Arg] and **4c** [**1**-Gly-Arg]). This irregular observation for **4c** implies that the relative thermodynamic stability between the [Se–Se] and [SeH,SeH] forms is controlled by the peptidyl conformations, which strongly affect the topological configuration between the Se atoms and guanidyl group in Arg and thus stabilize interactions, such as NH···Se hydrogen bonds in each redox state. Indeed, we previously suggested that [SeH,SeH] derived from **3** with a Pro spacer forms a γ-turn structure, closely approaching the imidazole ring to the Se atom to form an NH···Se hydrogen bond, improving the relative thermodynamic stability of the [SeH,SeH] form [28]. To obtain structural information on compound **4c** and its reduced ([SeH,SeH]) forms, circular dichroism (CD) spectra of the compounds were evaluated with **3** as the reference. Here, compound **4c′**, in which the selenol moieties in reduced **4c** were methylated, was used as a model compound in CD analysis because the [SeH,SeH] form was not purified or isolated because of its instability under aerial conditions. The results showed that the spectral profile of **4c** resembles that of **3**, suggesting that the structural properties of **3** with the Pro spacer and **4c** with Gly are similar (dashed lines in Figure 5). This indicates that the *E*°′_SeSe_ value of compounds are affected more by the structure of the chain-opened form ([SeH,SeH]) than by the [Se–Se] form. The CD spectra further suggested that the ring-opening of **4c** induced overall conformational changes at the peptide chain (red solid line in Figure 5), whereas a typical spectral profile indicating the γ-turn structure, as seen for open-chain form of **3** (black solid line in Figure 5), was not observed. Although further structural investigations of **4c** and **4c′** are needed, the [SeH,SeH] form of **4c** may possess a unique structural property by which the Se atom and guanidyl group are close to each other to stabilize the selenol form via non-covalent interactions, such as NH···Se hydrogen bonds.

### 3.3. Oxidative Folding of Lysozyme in the Presence of Diselenide Catalysts

**Figure 6:**
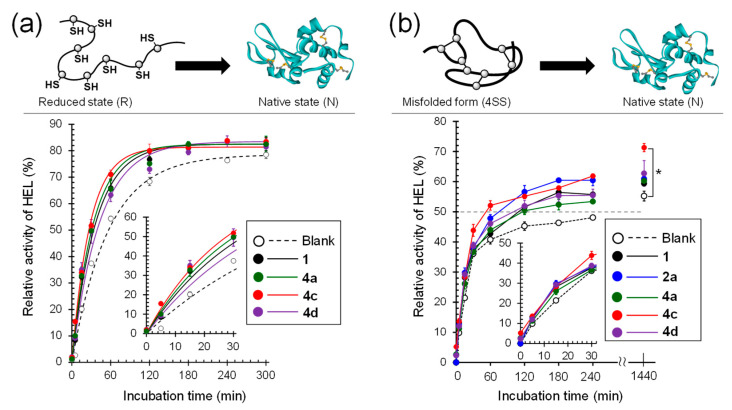
Comparison of the rates of oxidative folding of R^HEL^ and refolding of 4SS. Selected data are shown, and all other data for **1**-Gly-Xaa (series **4**) are shown in supporting information (Figure S1). Data are shown as means ± SEM (*n* = 3). (**a**) Enzymatic activity recovered during the oxidative folding of R^HEL^. The reaction conditions were [R^HEL^]_0_ = 10 μM, [GSH]_0_ = 1.0 mM, [GSSG]_0_ = 0.20 mM, [catalyst] = 0 or 20 μM, 37 °C, and pH 7.5 in the presence of 1 M urea. Circles and lines represent experimental data and simulations, respectively, which are drawn using the apparent rate constants (*k*). (**b**) Enzymatic activity recovered during the refolding of 4SS. The reaction conditions were [4SS]_0_ = 10 μM, [GSH]_0_ = 1.0 mM, [catalyst] = 0 or 20 μM, 37 °C, and pH 7.5 in the presence of 1 M urea.

To validate the capability of the compounds as folding catalysts, an oxidative folding assay using HEL without four intramolecular SS linkages (C6–C127, C30–C115, C64–C80, and C76–C94), i.e., reduced HEL (R^HEL^), was carried out, and the folding rates were compared among the employed catalysts. R^HEL^ was incubated in 100 mM Tris-HCl buffer solution containing GSH (1 mM) and GSSG (0.2 mM) in the presence or absence of a catalyst (20 μM) at pH 7.5 and 37 °C. The relative enzymatic activity of HEL recovered during the reaction was estimated after specific time points using a previously described protocol [32] and plotted against the incubation time (Figure 6a and Figure S1a). The result clearly showed that the enzymatic activity of HEL was gradually recovered with progression of the catalytic folding and implied that R^HEL^ was converted into a three-dimensionally folded state. Indeed, CD and HPLC analyses during the oxidative folding showed that the structural maturation of HEL progressed (Figure S2). The time courses of the enzymatic activity were fitted using a single exponential function [% activity = *A*_max_(1−e^−*kt*^)] [33], and the apparent rate constant (*k*) was estimated (Table 1). The maximal enzymatic activity recovered during oxidative folding (*A*_max_) was approximately 80% for all compounds, and no significant difference was observed among the employed catalysts. On the other hand, the *k* values observed when compound (**4**) with a Gly spacer was used as the catalyst were comparable to or lower than those of the corresponding compounds (**2**) without a Gly spacer, while the insertion of the Gly spacer obviously improved the capability of the oxidative folding catalyst for the compounds conjugated with Arg (i.e., **2c** vs. **4c**); in contrast, **4c** has a higher catalytic activity than the other compounds (Table 1 and Figure 6a). Considering the plausible catalytic mechanisms shown in Figure 2b, in principle, a compound with a higher *E*°′ value should show an enhanced oxidative folding velocity. However, the catalytic activity of the compounds did not exactly correlate with the *E*°′_SeSe_ values. Notably, the catalytic activity of **4c** (**1**-Gly-Arg; *E*°′_SeSe_ = −331 ± 3 mV) was comparable to or slightly higher than that of **2a** (**1**-His; *E*°′_SeSe_ = −304 ± 7 mV), which had the highest folding catalytic activity among the cyclic diselenides directly conjugated with a basic amino acid, whereas the *E*°′_SeSe_ of **4c** was lower than that of **2a**. This strongly indicates that the ability of compounds to serve as folding catalysts is not governed only by the thermodynamics of the Se–Se bond, and thus, there are important determinants for the catalytic activity besides the *E*°′_SeSe_ value (see Section 3.5).

**Table 1:** Summary of obtained parameters *^a^*.

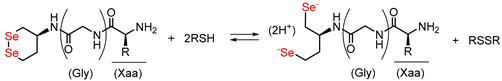								
Compound			*E*°′_SeSe_ [mV] *^b^*	*v*^0^_BPIns_ [μM min^−1^] *^c^*	R^HEL^ → N^HEL^		4SS → N^HEL^ *^d^*	
	Gly (or Pro)	Xaa			*A*_max_ [%] *^d^*	*k* [×10^−3^ min^−1^] *^e^*	*A*_max_ [%] *^d^*	*t*_50_ [min] *^f^*
**1***^g^*:	*None*	*None*	−368 ± 5	1.25 ± 0.03	81.1 ± 0.7	27.6 ± 0.6	59.4 ± 0.9	109 ± 4
**2a***^g^*:	*None*	His	−304 ± 7	2.45 ± 0.06	83.0 ± 1.3	32.7 ± 0.7	60.9 ± 0.7	74 ± 8
**3**:	Pro	His	−289 ± 8 *^h^*	ND *^i^*	82.8 ± 0.7 *^h^*	32.8 ± 1.4 *^h^*	60.1 ± 1.1	122 ± 17
**4*a***:	Gly	His	−356 ± 2 *^h^*	2.00 ± 0.14	82.5 ± 1.0 *^h^*	30.6 ± 0.9 *^h^*	60.3 ± 0.6	127.0 ± 26.2
**2b***^g^*:	*None*	Lys	−352 ± 2	1.94 ± 0.08	82.7 ± 3.2	29.8 ± 0.6	59.1 ± 0.1	107 ± 10
**4b**:	Gly	Lys	−352 ± 1	2.14 ± 0.03	83.2 ± 0.1	31.3 ± 1.8	53.7± 1.6	167 ± 9
**2c *^g^***:	*None*	Arg	−353 ± 1	2.64 ± 0.06	79.8 ± 0.4	30.9 ± 1.2	57.7 ± 0.1	105 ± 5
**4c**:	Gly	Arg	−331 ± 3	2.65 ± 0.05	81.4 ± 1.0	35.4 ± 1.8	72.1 ± 1.4	54 ± 9
**2d***^g^*:	*None*	Ala	−340 ± 2	2.22 ± 0.07	80.6 ± 0.3	28.3 ± 1.4	59.0 ± 0.7	101 ± 5
**4d**:	Gly	Ala	−361 ± 2 *^h^*	2.12 ± 0.05	83.5 ± 2.1 *^h^*	23.5 ± 2.3 *^h^*	62.8 ± 4.2	91 ± 6
No compound *^g^*			−	0.36 ± 0.02	78.6 ± 1.5	17.9 ± 0.6	55.2 ± 1.6	>240

*^a^* All data are shown as mean ± SEM (*n* = 3). *^b^* Diselenide reduction potentials were estimated by following a method reported in the literature [16]. *^c^*Initial velocity estimated from Figure S3 for catalytic SS-reduction of bovine pancreatic insulin (BPIns). *^d^* Maximal enzymatic activity of HEL recovered in the folding experiments. *^e^* Apparent rate constant obtained by fitting the experimental data (Figure 6a). *^f^* Time required to reach 50% recovery of HEL enzymatic activity in the refolding of 4SS. *^g,h^* Data reported in [18,28], respectively. *^i^* Not determined.

### 3.4. Repairing of Scrambled Lysozyme in the Presence of Diselenide Catalysts

Subsequently, to validate the ability of the compounds to repair misfolded (scrambled) proteins, refolding experiments using 4SS of HEL as a misfolded substrate [18], which has four non-native SS bonds in the molecule, was carried out in a buffer solution containing 1 mM GSH and 1 M urea at pH 7.5 and 37 °C in the presence or absence of a diselenide catalyst. The enzymatic activity of HEL recovered in the sample solutions was plotted against the reaction time (Figure 6b and Figure S1b). The results showed that the refolding phase could likely be divided into two phases: the fast and slow phases, during which the enzymatic activity of HEL was recovered by approximately 40% within 30 min and by 60–70% after the fast phase, respectively. Therefore, the effectiveness of the compounds as refolding catalysts was assessed by comparing the time (*t*_50_) required to recover 50% enzymatic activity because the experimental data obtained from the refolding of 4SS could not be fitted by the single exponential function (Table 1). Although the initial velocity observed for all compounds in the fast phase was almost the same, the *t*_50_ observed in the presence of **4c** (**1**-Gly-Arg) was the shortest compared to those observed in the presence of other compounds (Figure 6b and Table 1). Interestingly, **4c** also improved the recovered maximal enzymatic activity (*A*_max_) by approximately 10% over the yields observed in the presence of other compounds (Figure 6b and Table 1).

**Figure 7:**
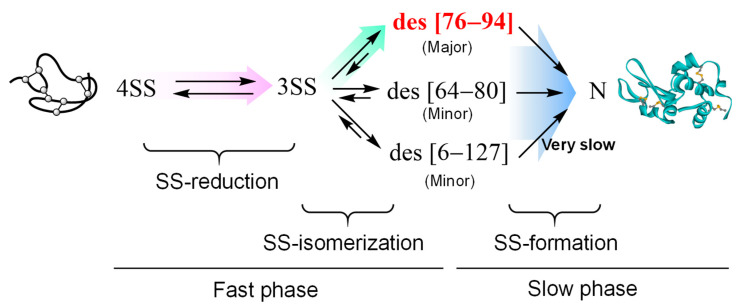
Putative major refolding pathways of scrambled HEL (4SS) to N at physiological temperature.

Considering the oxidative folding pathways of HEL, which have been well characterized by several groups [2], to refold from 4SS to N, 4SS is initially reduced to 3SS, and the generated 3SS is subsequently isomerized to des intermediates, which are 3SS species lacking one (C76–C94, C64–C80, or C6–C127) of the four SS bonds found in N and have a stable native-like structure (Figure 7). Finally, by forming the final SS bond in the des intermediates, N is reproduced. By applying a temperature close to the biological condition, des (76–94), which possesses 50% enzymatic activity with respect to N and is a kinetically trapped intermediate in the folding pathway, is generated as a major metastable precursor of N [29]. Thus, the fast initial phase and slow later phase observed in Figure 6b should mainly correspond to the generation processes of des (76–94) involved in SS-reduction and SS- isomerization and its slow conversion into N, respectively.

To evaluate the relationship between the capability of the compounds as SS-reductase-like catalysts and refolding initial velocity, catalytic SS-reduction using bovine pancreatic insulin (BPIns) as a substrate was carried out as previously described [16,18]. The initial velocities (*v*_0_^Ins^) for SS-reduction observed in the presence of the compounds (series **2** and **4**) with amino acids and dipeptides were 1.5–2.0-fold higher than that in the presence of the parent compound (**1**) (Table 1 and Figure S3). However, the initial folding velocities observed for all compounds, including compound **1**, were similar (Figure 6b), suggesting that SS-reductase activity is not a major factor governing the efficiency of the initial fast phase in 4SS refolding and that diselenides contribute more to SS-isomerization for the conversion of 3SS into des intermediates. However, no correlation was found between the reaction-promoting abilities of compounds in the fast phase and their *E*°′_SeSe_ values, which is a key indicator of the ability of the compounds to function as SS-isomerase-like catalysts. Similarly, an exact correlation between the final folding yields (*A*_max_) as well as the velocity (*t*_50_) and *E*°′_SeSe_ values, which also represent the oxidizing power of the compounds, were not observed again in the later slow phase, which involves the SS-formation of the kinetically trapped des intermediate des (76–94).

### 3.5. Suppressive Capbility of Diselenide Compounds against Protein Aggregation

**Figure 8:**
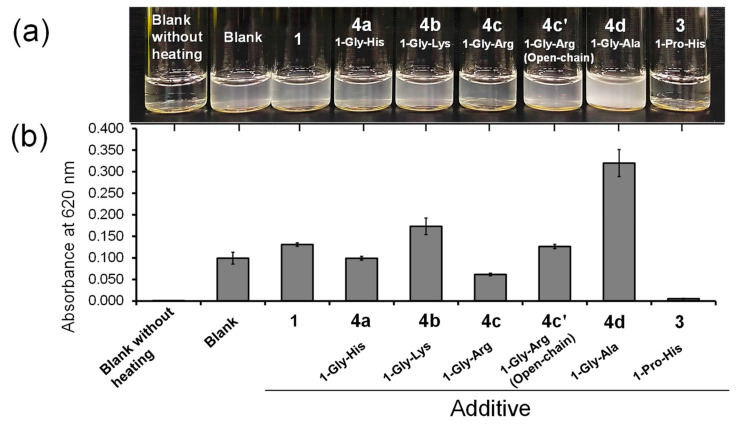
Thermal denaturation-induced aggregation of HEL. (**a**) Photograph of HEL (100 μM) in a buffer solution containing no compound or 0.5 mM compound, 0.28 M NaCl, and 1 mM EDTA at pH 7.5 and 23 °C obtained after heat-denaturation at 90 °C for 10 min. (**b**) Light scattering at 620 nm due to generation of visible precipitation of HEL aggregates. Reaction conditions were the same as in (**a**).

In the oxidative folding and refolding experiments, there was an approximate tendency for compounds with higher *E*°′_SeSe_ values to exert higher catalytic activity, indicating that the thermodynamic stability of the Se–Se bond would be one of key determinants of the catalytic abilities. However, the correlation between these factors was not apparent. Indeed, compound **4c** exhibited the best catalytic activity among the cyclic diselenide compounds employed in both oxidative folding of R^HEL^ and refolding of 4SS. One factor that should be further considered is intermolecular protein–compound interactions, which increase the frequency of contact between the compound and protein molecule. If a compound can easily interact and associate with proteins, it can effectively suppress oligomerization and aggregation through associations between structurally immature proteins via intermolecular interactions such as electrostatic and hydrophobic interactions. Therefore, the aggregation induced by thermal denaturation of native HEL (N; 100 μM) was conducted in the presence or absence of the compounds (0 or 0.5 mM), as previously described [18]. Although aggregation occurred in all sample solutions, in the presence of **4c** (**1**-Gly-Arg), the turbidity of the solution was slightly lower than that in the other samples containing a Gly-Xaa dipeptide conjugate (i.e., series **4**) (Figure 8a). In contrast, **4d** (**1**-Gly-Ala) promoted aggregation rather than suppression. When the turbidity of the solutions was measured by light scattering at 620 nm due to the aggregates, **4c** suppressed the aggregation of HEL most effectively and showed the highest association with the denatured proteins among the compounds with a Gly spacer (Figure 8b). In principle, the folding yield should not be changed with or without catalysts because a catalyst does not alter the heat of the reaction but rather reduces the activation energy. However, addition of a catalytic amount of **4c** to the refolding solution increased the final yield of active HEL (Figure 6b), indicating that **4c** suppressed the undesired formation of invisible oligomers during refolding. Interestingly, the aggregation suppression activity of **4c′** was lower than that of **4c**, suggesting that an open-chain form is less favored than a closed-chain diselenide form as the molecular structure of the aggregation suppressor. However, it was previously reported that compound **2a** completely prohibited the thermal denaturation-induced aggregation of HEL under the same conditions as those described in Figure 8. Similarly, compound **3**, which showed a lower catalytic activity than **4c** in both oxidative folding of R^HEL^ and refolding 4SS (Table 1), effectively inhibited HEL aggregation (Figure 8). Thus, the associating capabilities of **2a** and **3** with denatured proteins should be higher than that of **4c**, which is inconsistent with the order of the catalytic activity (**4c** [*E*°′_SeSe_ = −331 mV] ≥ **2a** [*E*°′_SeSe_ = −304 mV] ≈ **3** [*E*°′_SeSe_ = −289 mV]).

### 3.6. Future Prospects

Herein, we mainly focused on the diselenide thermodynamics and affinity of compounds with substrate proteins, although the kinetics of reducing Se–Se bonds and p*K*_a_ value (nucleophilicity) of the selenol groups in the active [SeH,SeH] forms are also key factors for the catalytic activity of the oxidative folding catalyst. In addition, the reactivity of [Se–Se] and [SeH,SeH] forms with GSH/GSSG as effective redox reagents during the reactions should be considered. Therefore, to design more effective folding catalysts, it is necessary to comprehensively investigate the physicochemical properties of compounds, including their association with proteins, selenol p*K*_a_, and activation kinetics. In addition, at present, the PDI-like activity of compounds was investigated by using only HEL as a model protein. Measuring the substrate scope of the structurally optimized catalyst (**4c**) by applying it as a catalyst in the oxidative folding of various SS-containing proteins may provide useful information in the field of protein engineering for artificial protein design and synthesis. Furthermore, to investigate the possibility of pharmacological application of the compounds, their suppressive ability against pathogenic aggregation using other model proteins, which transform into amyloid-type aggregates, should be evaluated.

## 4. Conclusions

(*S*)-1,2-Diselenane-4-amine (**1**) conjugated with Gly-Arg dipeptide (**4c**) was developed as an effective catalyst for oxidative folding of a reduced protein and refolding of a scrambled protein. Importantly, the catalytic activity of **4c** was comparable to or slightly higher than that of **1**-His (**2a**), which enhanced the velocities of oxidative folding and refolding among cyclic diselenide compounds directly conjugated with basic amino acids. Although insertion of a Gly spacer into the basic amino acid conjugates (series **2**) generally did not affect or slightly decreased the catalytic activities, when the terminal amino acid was Arg, the catalytic activity was abnormally improved with an increasing *E*°′_SeSe_ value. However, the precise correlation of the *E*°′_SeSe_ value with the catalytic activity of the compounds was not determined; the compounds should perform catalysis via the mechanisms shown in Figure 2b, which are similar to those of PDI. The thermal denaturation-induced aggregation assay showed that compound **4c** reasonably suppressed aggregation and thus suggested that **4c** readily associated with denatured proteins to inhibit intermolecular protein–protein interactions. Thus, not only the thermodynamics of Se–Se bonds but also other critical factors, including the ability of compounds to associate with immature proteins, should be considered when designing effective PDI-like catalysts.

From the perspective of the future application of compounds, structurally optimized compound (**4c**) is a promising catalyst for promoting the folding process in preparations of peptide-based drugs such as oxytocin and insulin. In addition, it has recently been reported that dysfunction of PDI causes ER stress, resulting in cell death and critical conditions such as Alzheimer’s and Parkinson’s diseases. Although simple model experiments in vitro were performed in this study, biological studies using cells and model animals are needed before applying the compound as an alternative molecule of PDI.

## Data Availability

Not applicable.

## Supplementary Materials

The following are available online at https://www.mdpi.com/article/10.3390/biology10111090/s1, Supplemental procedures for synthesis of **4c′**, ^1^H, ^13^C, and ^77^Se NMR spectra for compounds **4b**, **9**, and **4c′**, Figure S1: Comparison of the rates for oxidative folding of R^HEL^ and refolding of 4SS, Figure S2: CD spectroscopic and HPLC analyses during the oxidative folding of HEL, Figure S3: Disulfide reductase-like activity of the cyclic diselenides.
