# Supplementary material for: Abnormal Enhancement of Protein Disulfide Isomerase-like Activity of a Cyclic Diselenide Conjugated with a Basic Amino Acid by Inserting a Glycine Spacer
Source: Biology (Basel). 2021 Oct 24;10(11):1090. doi: 10.3390/biology10111090 (PMC8615077; doi:10.3390/biology10111090)
Supplement: Supplementary file 1 [file biology-10-01090-s001.zip › biology-1378158-supplementary.pdf]

Supporting information

Abnormal Enhancement of Protein Disulfide Isomerase-like Activity of a Cyclic Diselenide Conjugated with a Basic Amino Acid by Inserting a Glycine Spacer

Rumi Mikami[1], Shunsuke Tsukagoshi[1], and Kenta Arai[1]*

[1]Department of Chemistry, School of Science, Tokai University, Kitakaname, Hiratsuka-shi, Kanagawa 259-1292, Japan

Contents

1. Synthesis of compound 4c'

*Synthesis of (S)-2-amino-N-(1,4-bis(methylselanyl)butan-2-yl)acetamide (**9**)*

*Synthesis of (S)-2-amino-N-(2-(((S)-1,4-bis(methylselanyl)butan-2-yl)amino)-2-oxoethyl)-5-guanidinopentanamide deTFA salt (**4c'**)*

2. NMR spectra

Compound 4b *(S)-N-(2-(((S)-1,2-diselenan-4-yl)amino)-2-oxoethyl)-2,6-diaminohexanamide di-TFA salt*

Compound 4c *(S)-N-(2-(((S)-1,2-diselenan-4-yl)amino)-2-oxoethyl)-2-amino-5-guanidinopentanamide di-TFA salt*

Compound 9 *(S)-2-Amino-N-(1,4-bis(methylselanyl)butan-2-yl)acetamide TFA salt*

Compound 4c' *(S)-2-Amino-N-(2-(((S)-1,4-bis(methylselanyl)butan-2-yl)amino)-2-oxoethyl)-5-guanidi nopentanamide di-TFA salt*

3. Supplemental Figures

Figure S1 Comparison of the rates for oxidative folding of R^{HEL} and refolding of 4SS.

Figure S2 CD and HPLC analyses during the oxidative folding of HEL.

Figure S3 Disulfide reductase-like activity of the cyclic diselenides.

1. Synthesis of compound 4c'

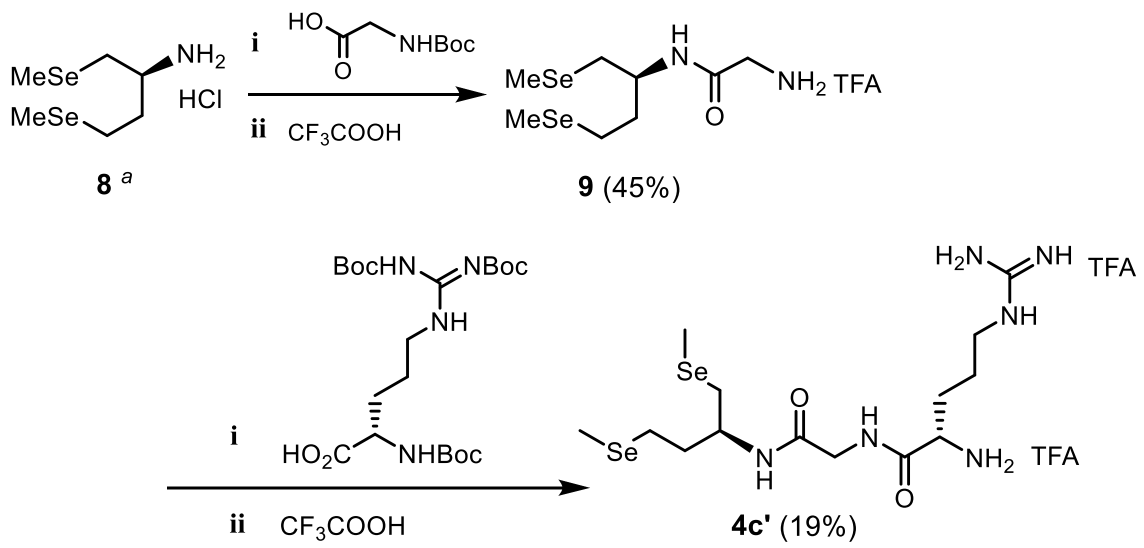

Scheme S1: Synthetic routes for compound **4c'**. Reaction conditions: (i) PyBOP, DIPEA, DMF, 25 °C for 16 h; (ii) CH_2Cl_2, 25 °C, 4 h. [a] Compound **8** was prepared by following the previous methods [Ref 18; K. Arai, *et al.*, *Chem. Asian J.* **2014**, *9*, 3464–3471] with slight modifications.

*Synthesis of (S)-2-amino-N-(1,4-bis(methylselanyl)butan-2-yl)acetamide TFA salt (compound **9**)*

Compound **8** (20.6 mg, 69.7 μmol) was added to a solution of Boc-protected Gly (24.4 mg, 139.4 μmol, 2 eq) dissolved in anhydrous DMF (1.5 mL). PyBOP (72.5 mg, 139.4 μmol, 2 eq) and DIPEA (35.4 μL, 202.8 mmol, 4 eq) were added to the solution. The mixture was stirred for 16 h at room temperature under argon atmosphere. The solution was then diluted with water (15 mL) and the solution was extracted with dichloromethane (15 mL×3). The organic phase was washed with saturated aqueous NH_4Cl (20 mL×1), water (20 mL×1), and brine (20 mL×1). The obtained organic phase was then dried by passing through a column-type phase separator (ISOLUTE®) to completely remove a residual aqueous phase and concentrated under vacuum. The residual material as a crude product was purified by silica gel column chromatography (EtOAc/*n*-hexane, 3:1 [*v*:*v*]) to give Boc-protected form of **9**. To deprotect the Boc group, to the previous compound in dichloromethane (0.8 mL) was added with TFA (0.8 mL) at 0 °C and stirred for 30 min. Then, the mixture was further stirred at room temperature for 4 h. The resulting solution was evaporated to remove the solvent and TFA. The residual yellow oil was purified by an HPLC equipped a reverse-phase column (Tosoh TSKgel ODS-100Z 4.6×150 mm). The column was equilibrated with 100:0 (v/v) mixture of 0.1% TFA in water (eluent A) and 0.1% TFA in acetonitrile (eluent B) at a flow rate of 1.0 mL/min. After injection of the sample solution, a solvent gradient (a ratio of eluent B linearly increased from 0% to 50% in 0–20 min) was applied. The fraction for the target compounds was collected and lyophilized to give compound **9** as a coreless oil. Yield: 13.6 mg (45%); ^{1}H NMR (500 MHz, D_2O): δ 4.10–4.05 (m, 1H), 3.73 (s, 2H), 2.75–2.56 (m, 2H), 2.55–2.42 (m, 2H), 1.98–1.86 (m, 7H), 1.82–1.75 (m, 1H) ppm, ^{13}C NMR (125.8 MHz, D_2O): δ 166.6, 49.9, 40.4, 34.1, 29.5, 20.9, 4.3, 3.4 ppm; ^{77}Se NMR (95.4 MHz, D_2O): δ 61.8, 36.7 ppm; HRMS (ESI-TOF) m/z: $[M+H–TFA]^+$ calcd for $C_8H_{19}N_2OSe_2^+$, 318.9822; found, 318.9831.

*Synthesis of (S)-2-amino-N-(2-(((S)-1,4-bis(methylselanyl)butan-2-yl)amino)-2-oxoethyl)-5-guanidinopentanamide deTFA salt (compound **4c′**)*

To compound **9** (26.2 mg, 60.9 μmol) in DMF (1.5 mL) was added with Boc-L-Arg(Boc)$_2$ (57.8 mg, 121.8 μmol, 2 eq). PyBOP (63.4 mg, 121.8 μmol, 2 eq) and DIPEA (42.4 μL, 243.6 mmol, 4 eq) were then added to the solution and the resulting mixture solution was stirred for 16 h at room temperature under argon atmosphere. The mixture solution was then directly passed through a silica gel column equilibrated with a mixture solvent of EtOAc/*n*-hexane (5:1 [*v*:*v*]) to remove impurities and DMF and the fractions containing a Boc-protected dipeptide conjugate were collected, combined, and evaporated. The obtained residual material was further purified by GPC and the collected fraction containing the Boc-protected target compound was collected and evaporated. To deprotect the Boc groups, to the previous compound in dichloromethane (0.8 mL) was added with TFA (0.5 mL) at 0 °C and stirred for 30 min. Then, the mixture was further stirred at room temperature for 5 h. The resulting solution was evaporated to remove the solvent and TFA. The residual yellow solid was purified by RP-HPLC. The HPLC conditions were same as those applied for compound **9**. The fraction for the target compound was collected and lyophilized to give compound **4c′** as a colorless oil/solid. Yield: 8.2 mg (19%); ^{1}H NMR (500 MHz, D_2O): δ 4.06–4.01 (m, 1H), 3.96 (t, *J* = 6.4 Hz, 1H), 3.86 (q, *J* = 16.6 Hz, 2H), 3.14 (t, *J* = 6.6 Hz, 1H), 2.72–2.68 (m, 1H), 2.58–2.49 (m, 2H), 2.45–2.39 (m, 1H), 1.95–1.73 (m, 4H), 1.91 (s, 3H), 1.88 (s, 3H), 1.66–1.52 (m, 2H) ppm; ^{13}C NMR (125.8 MHz, D_2O): δ 170.3, 169.9, 156.7, 52.7, 49.7, 42.3, 40.4, 34.1, 29.6, 27.9, 23.4, 21.0, 4.2, 3.3 ppm; ^{77}Se NMR (95.4 MHz, D_2O): δ 61.2, 37.9 ppm; HRMS (ESI-TOF) m/z: $[M+H–2TFA]^+$ calcd for $C_{14}H_{31}N_6O_2Se^{2+}$, 475.0833; found, 475.0852.

2. NMR spectra

Compound 4b:

(S)-N-(2-(((S)-1,2-Diselenan-4-yl)amino)-2-oxoethyl)-2,6-diaminohexanamide di-TFA salt

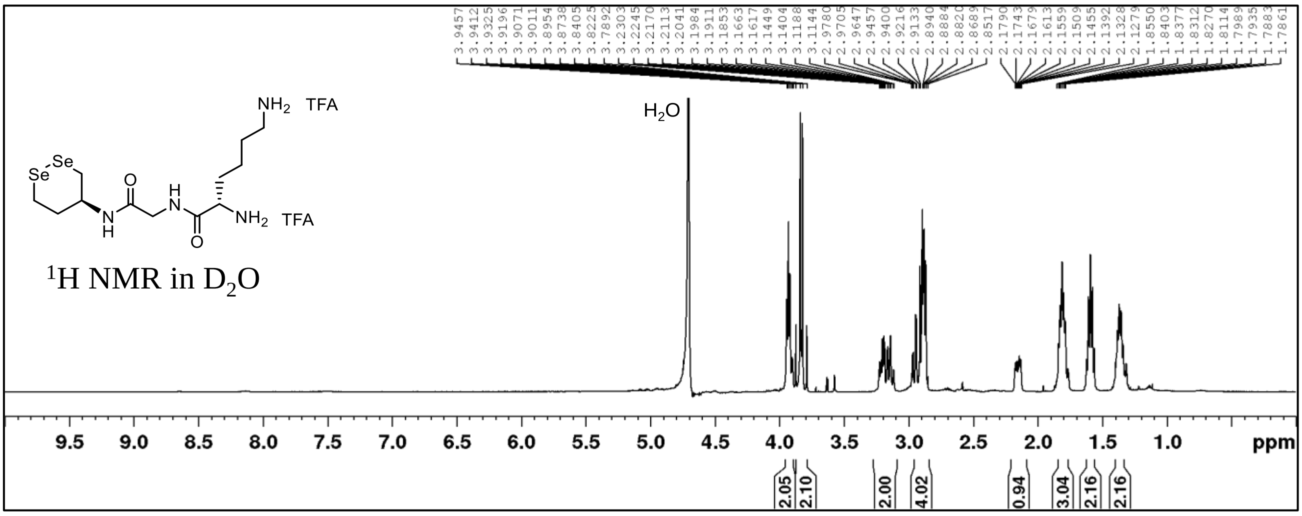

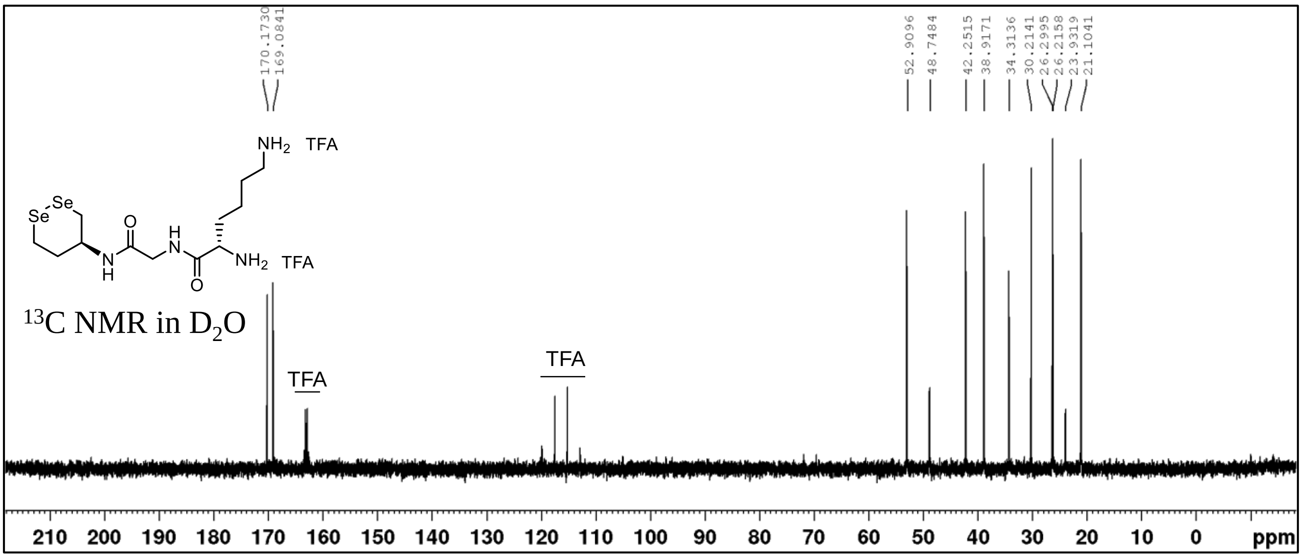

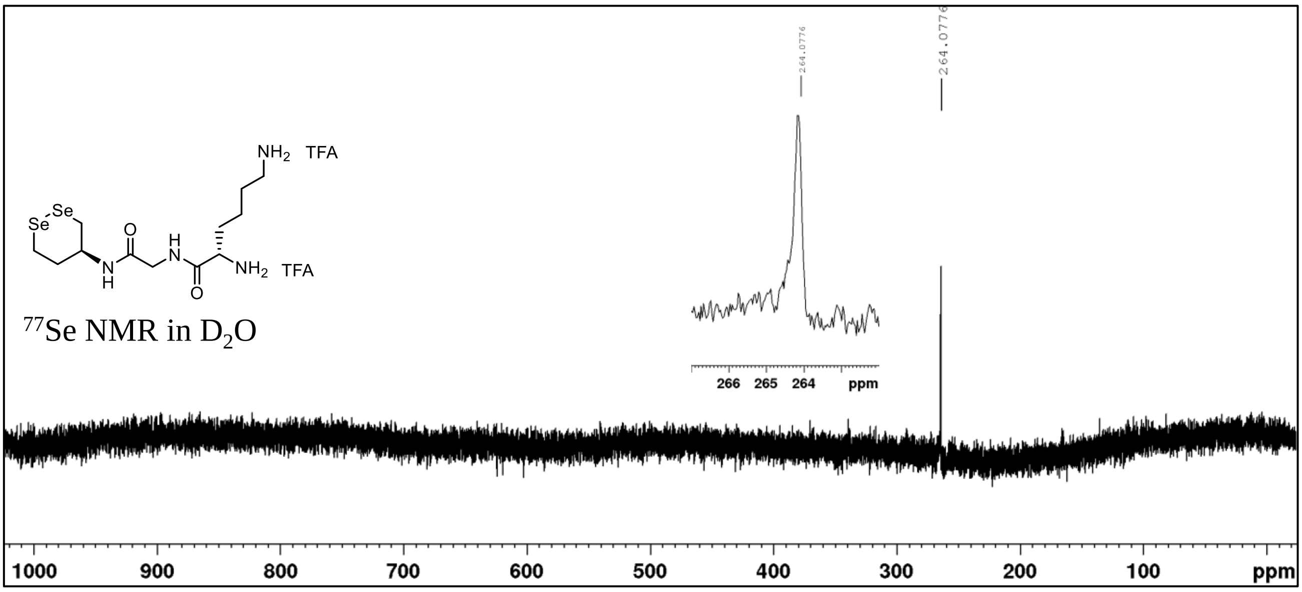

Compound 4c

(S)-N-(2-(((S)-1,2-Diselenan-4-yl)amino)-2-oxoethyl)-2-amino-5-guanidinopentanamide di-TFA salt

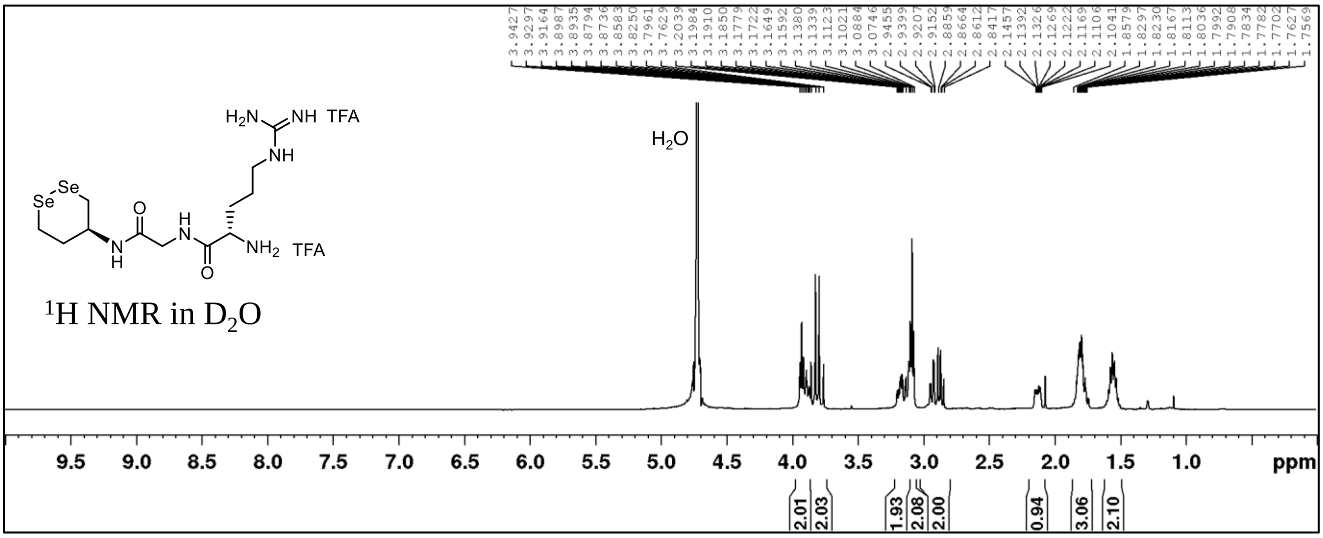

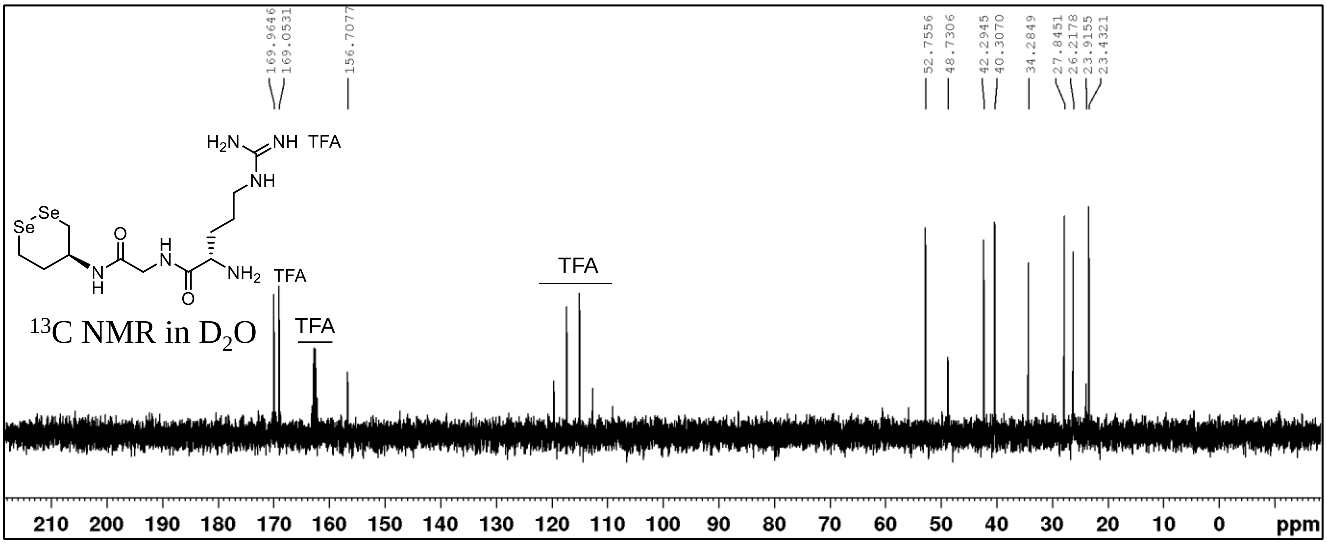

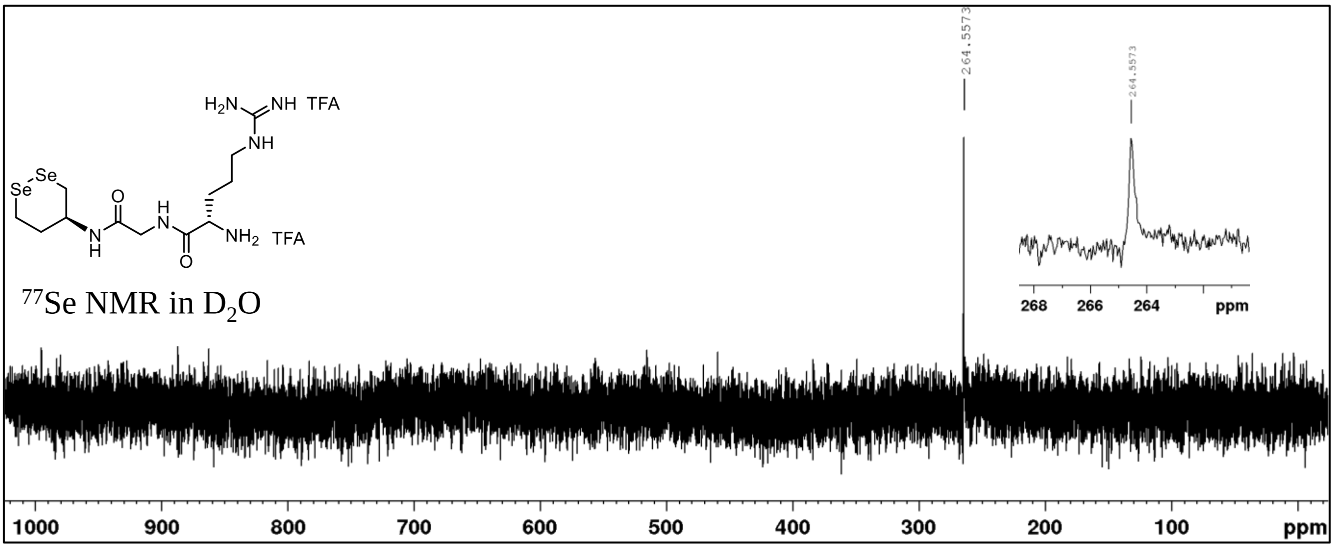

Compound 9

(S)-2-Amino-N-(1,4-bis(methylselanyl)butan-2-yl)acetamide TFA salt

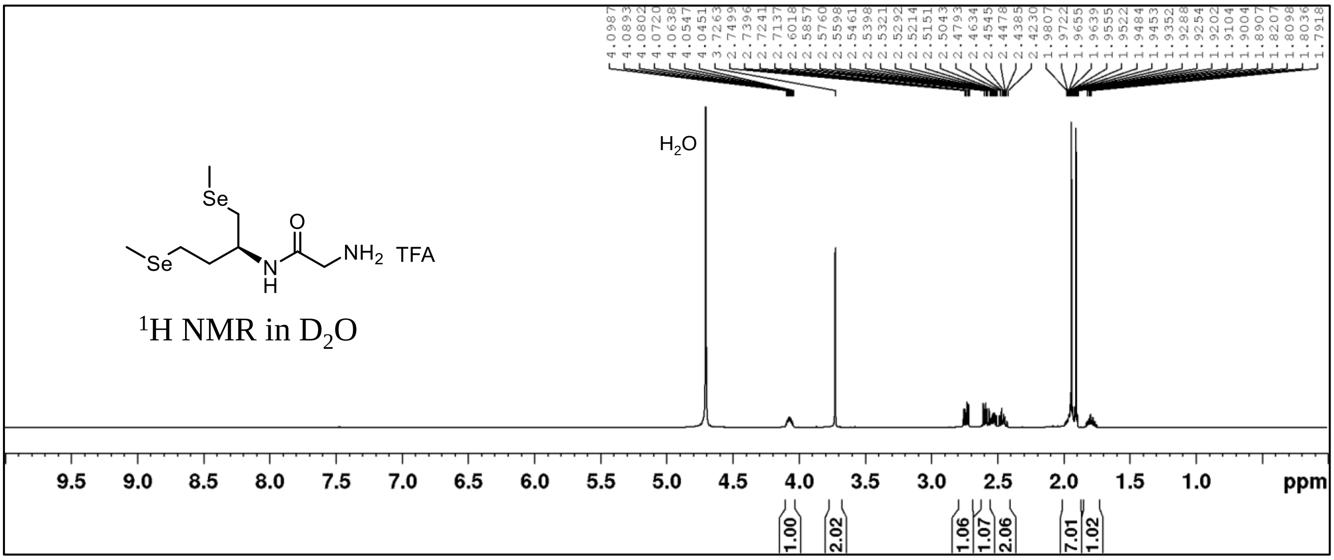

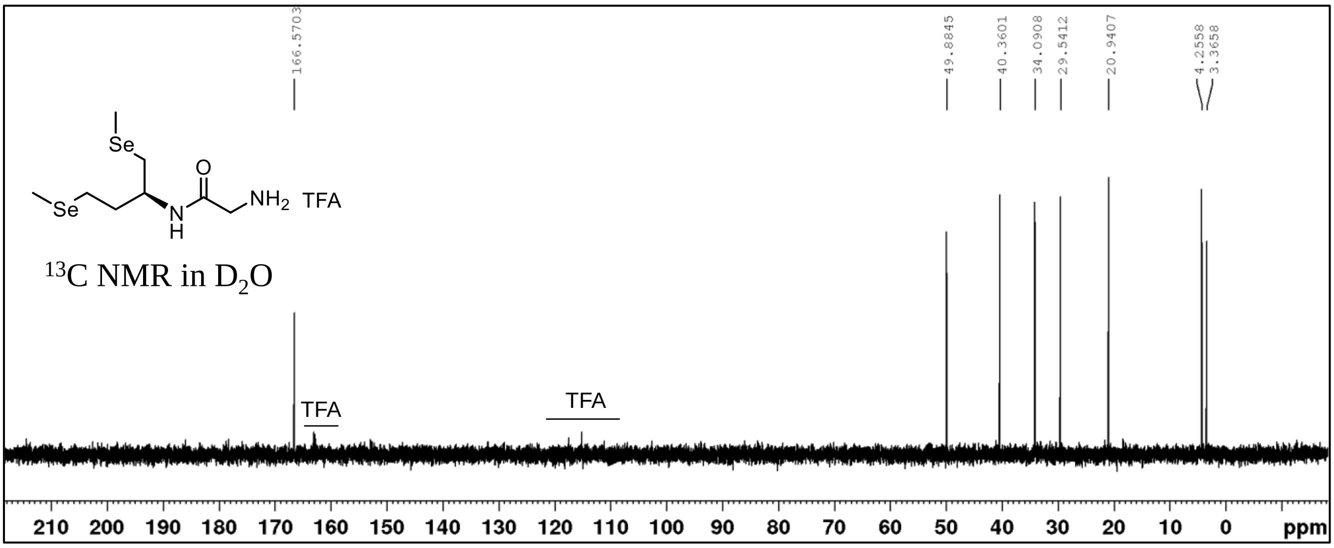

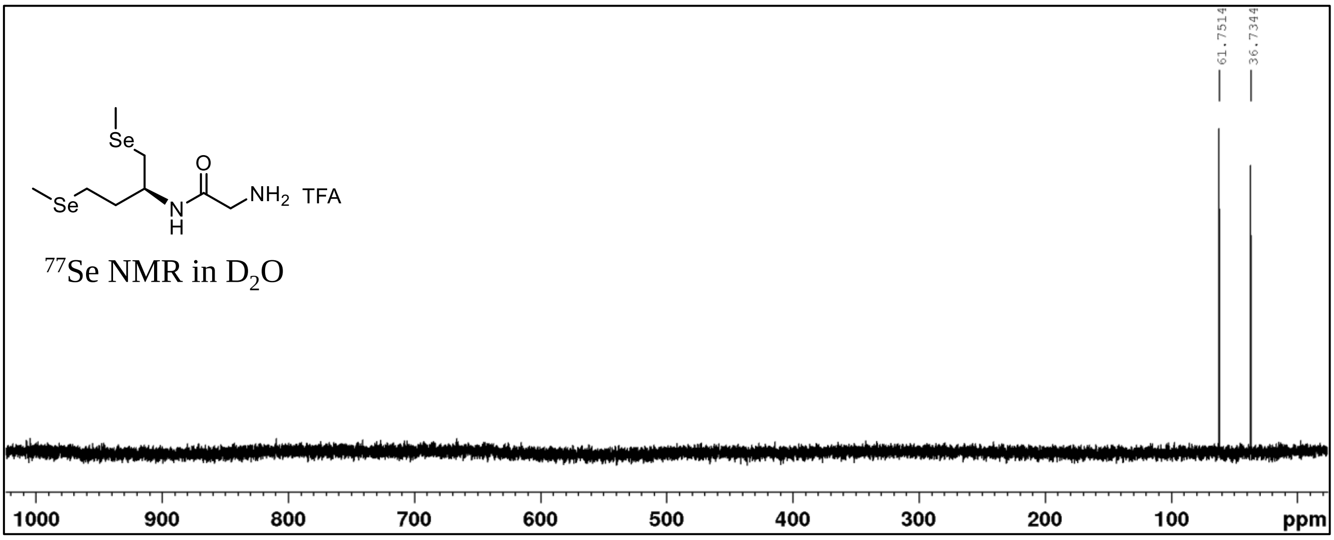

Compound 4c'

(S)-2-Amino-N-(2-(((S)-1,4-bis(methylselanyl)butan-2-yl)amino)-2-oxoethyl)-5-guanidinopentanamide di-TFA salt

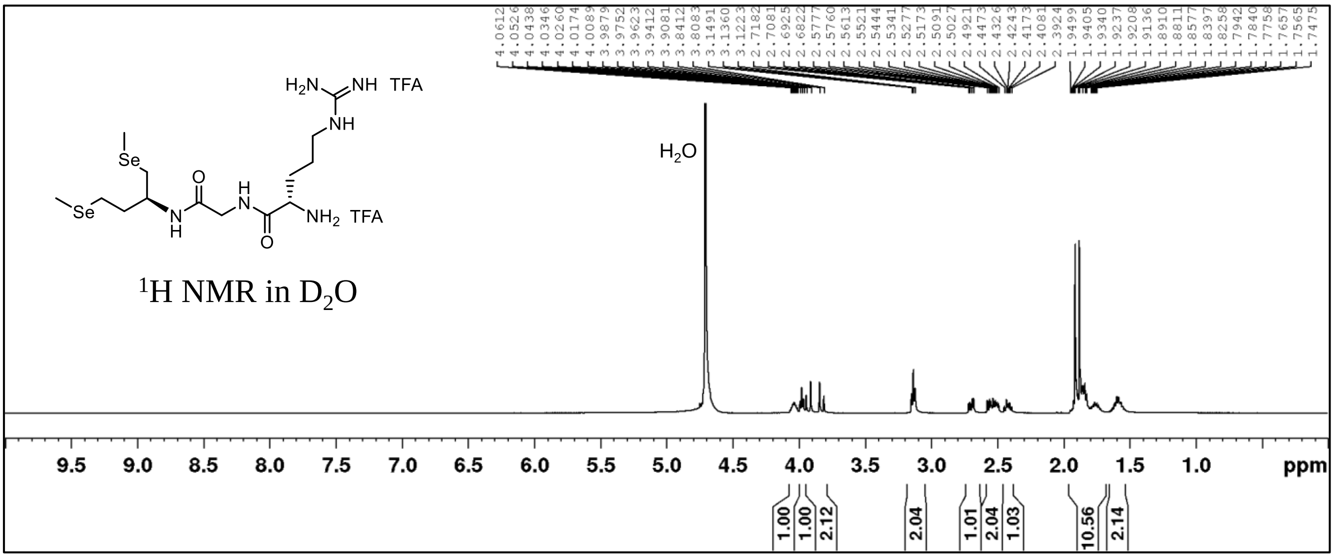

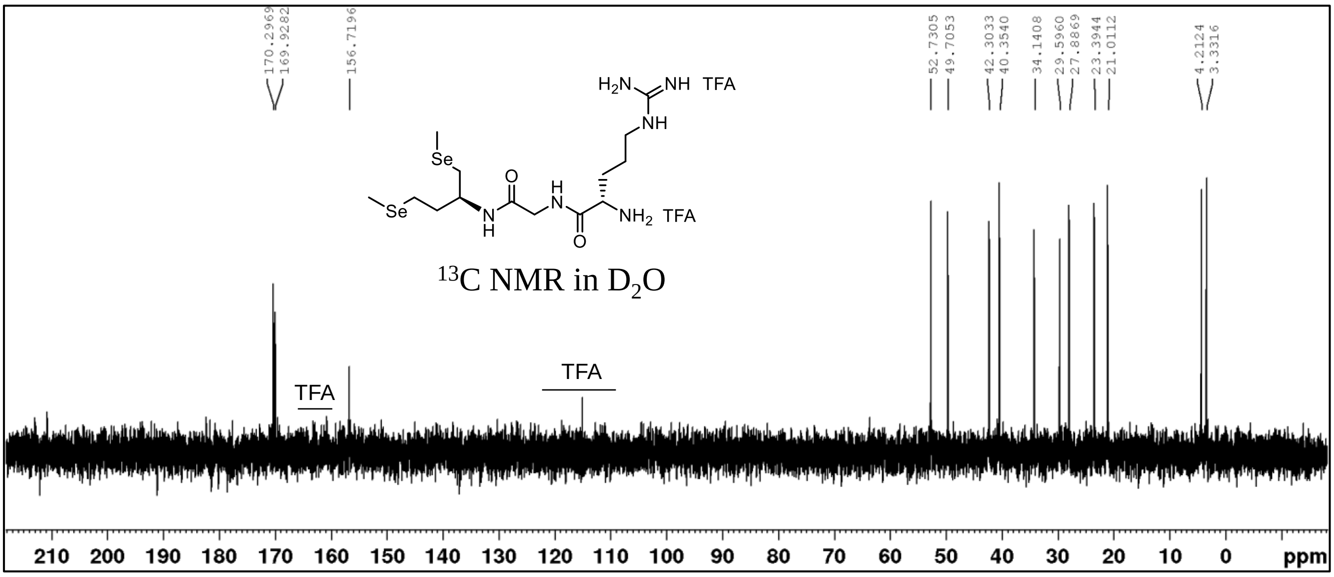

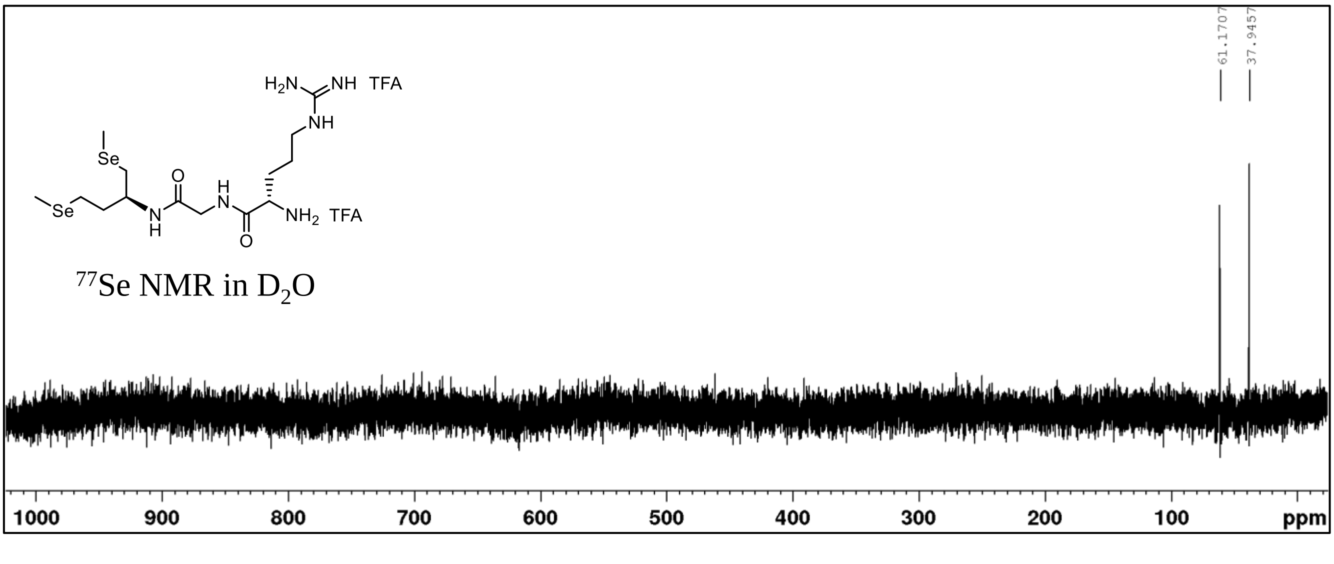

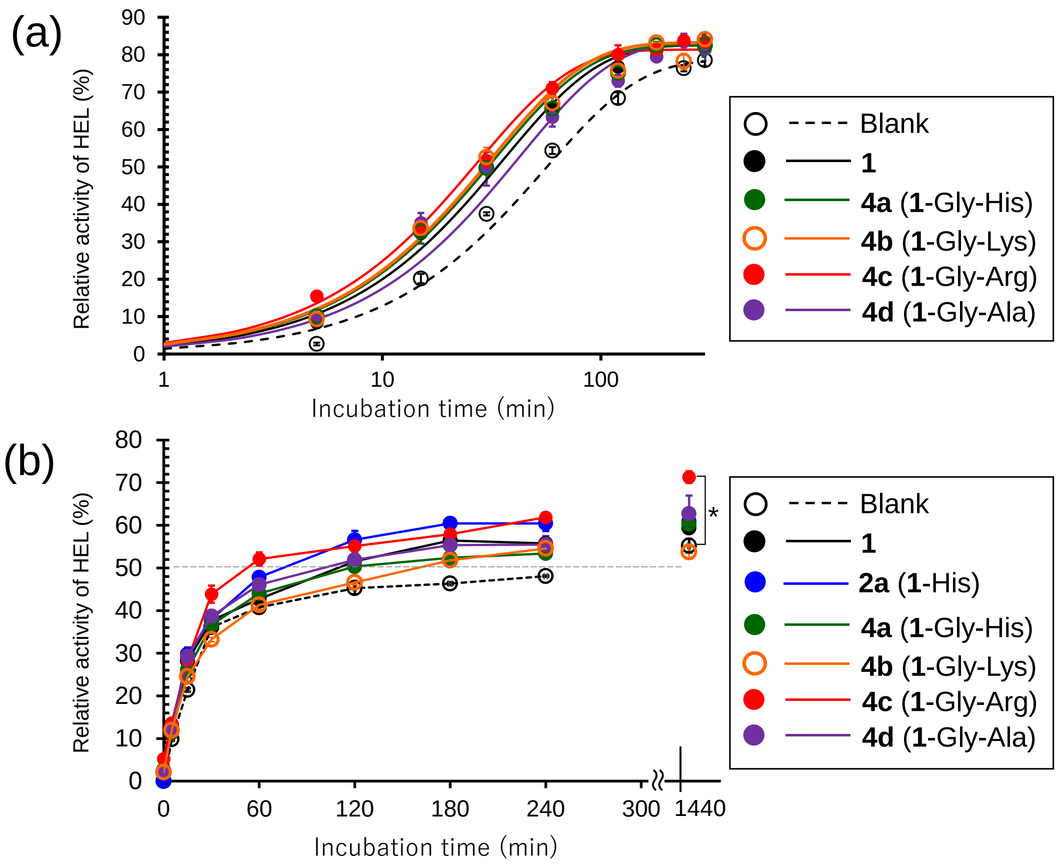

Figure S1. Comparison of the rates for oxidative folding of R^{HEL} and refolding of 4SS. Data are shown as means ± SEN (n=3). (**a**) Enzymatic activity recovered during the oxidative folding of R^{HEL}. The reaction conditions were same as those in Figure 6a. (**b**) Enzymatic activity recovered during the refolding of 4SS. The reaction conditions were same as those in Figure 6b.

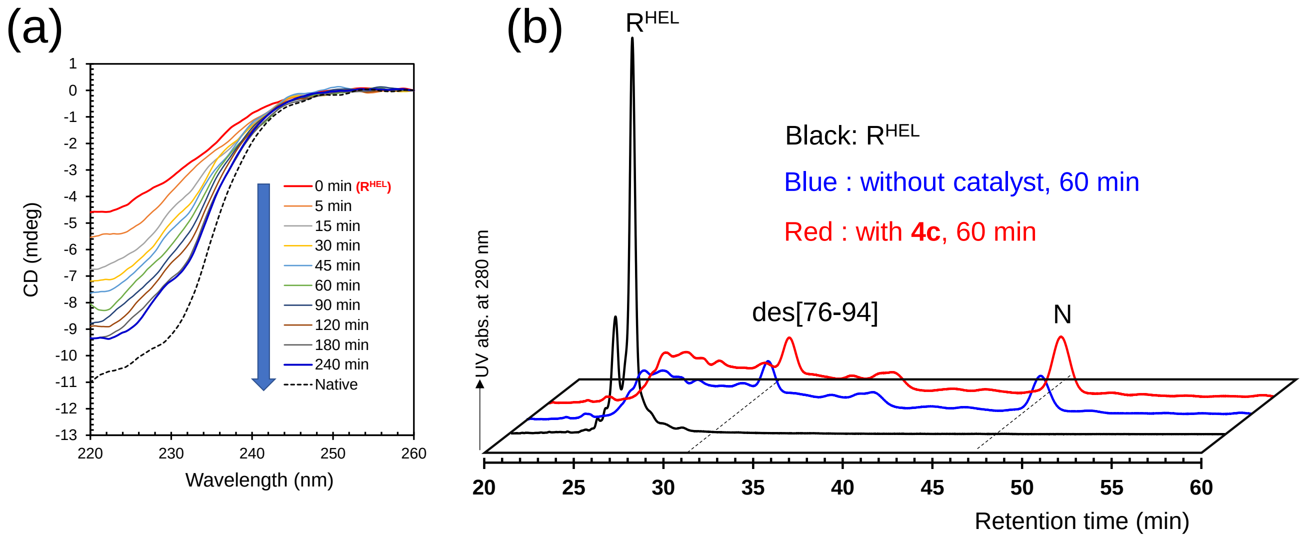

Figure S2. CD and HPLC analyses during the oxidative folding of HEL. The experiments were performed by following the previous literature [18]. (**a**) CD spectral changes observed during the oxidative folding of HEL in the presence of **4c**. Reaction conditions were $[R^{HEL}]_0 = 10$ µM, $[GSH]_0 = 1.0$ mM, $[GSSG]_0 = 0.20$ mM, [**4c**] = 20 µM, 37°C, and pH 6.9 in the presence of 1 M urea. (**b**) HPLC chromatograms obtained from the catalytic oxidative folding of R^{HEL} in the absence or presence of **4c**. The reaction conditions were the same as those in (**a**).

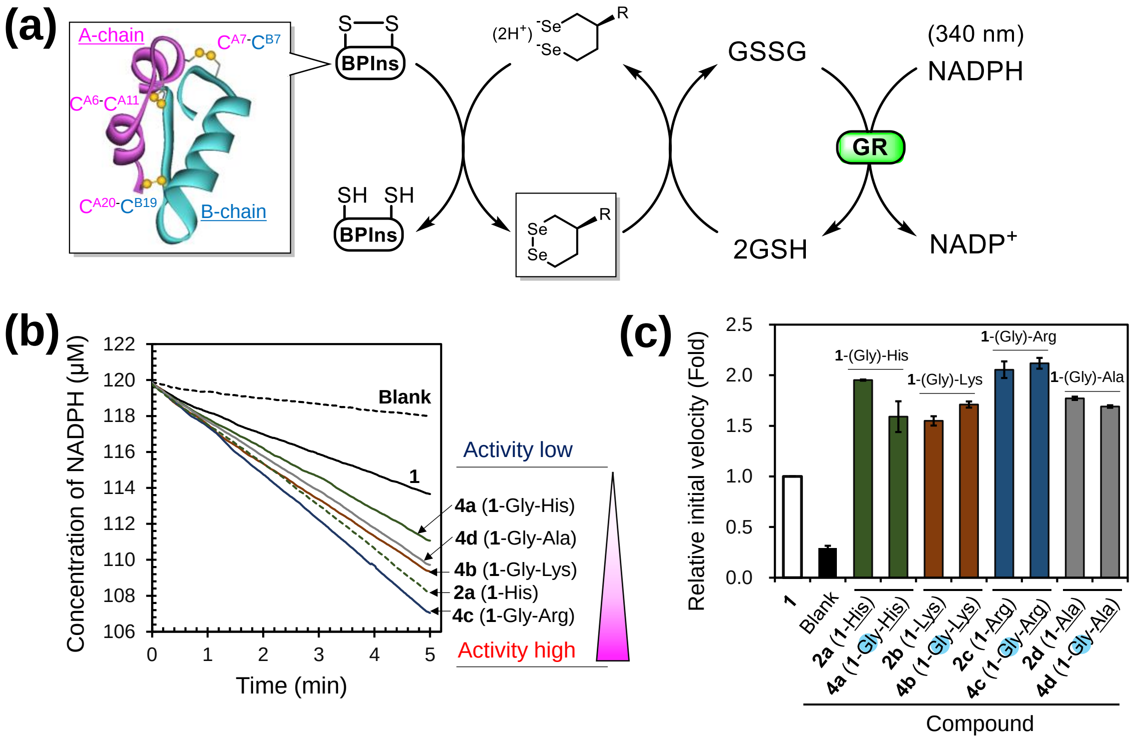

Figure S3. Disulfide reductase-like activity of the cyclic diselenides. (**a**) Catalytic SS reduction of bovine pancreatic insulin (BPIns; PDB code: 2bn3) in the absence or presence of diselenide compounds as catalysts together with NADPH, GSH, and GR. [see Ref 16 for the experimental details] (**b**) UV absorbance changes at 340 nm due to the consumption of NADPH during the SS-reduction of BPIns. The reaction conditions were $[\mathrm{BPIns}]_0$ = 60 μM, $[\mathrm{GSH}]_0$ = 3.7 mM, $[\mathrm{NADPH}]_0$ = 0.12 mm, and [GR]=4 unit mL^{-1}, together with [catalyst] = 30 μM at 25 °C in 200 mM phosphate buffer solution containing 5 mM EDTA at pH 7.5. (**c**) Relative initial velocity for the SS-reduction (in folds) in the presence of **1**-His or **1**-Gly-Xaa compared to that in the presence of **1** as the parent compound. All data are shown as means ± SEMs (n=3).
